# The Dark Side of Nosocomial Infections in Critically Ill COVID-19 Patients

**DOI:** 10.3390/life13061408

**Published:** 2023-06-17

**Authors:** Carmelo Biondo, Elena Ponzo, Angelina Midiri, Giuseppe Bernardo Ostone, Giuseppe Mancuso

**Affiliations:** Department of Human Pathology, University of Messina, 98125 Messina, Italy; cbiondo@unime.it (C.B.); elena.ponzo@studenti.unime.it (E.P.); amidiri@unime.it (A.M.); giuseppebernardo.ostone@studenti.unime.it (G.B.O.)

**Keywords:** COVID-19, co-infection, secondary infection, multidrug resistance, alternative strategies

## Abstract

Coronavirus disease 2019 (COVID-19) is a potentially serious acute respiratory infection caused by Severe Acute Respiratory Syndrome Coronavirus 2 (SARS-CoV-2). Since the World Health Organization (WHO) declared COVID-19 a global pandemic, the virus has spread to more than 200 countries with more than 500 million cases and more than 6 million deaths reported globally. It has long been known that viral respiratory tract infections predispose patients to bacterial infections and that these co-infections often have an unfavourable clinical outcome. Moreover, nosocomial infections, also known as healthcare-associated infections (HAIs), are those infections that are absent at the time of admission and acquired after hospitalization. However, the impact of coinfections or secondary infections on the progression of COVID-19 disease and its lethal outcome is still debated. The aim of this review was to assess the literature on the incidence of bacterial co-infections and superinfections in patients with COVID-19. The review also highlights the importance of the rational use of antibiotics in patients with COVID-19 and the need to implement antimicrobial stewardship principles to prevent the transmission of drug-resistant organisms in healthcare settings. Finally, alternative antimicrobial agents to counter the emergence of multidrug-resistant bacteria causing healthcare-associated infections in COVID-19 patients will also be discussed.

## 1. Introduction

**Figure 1:**
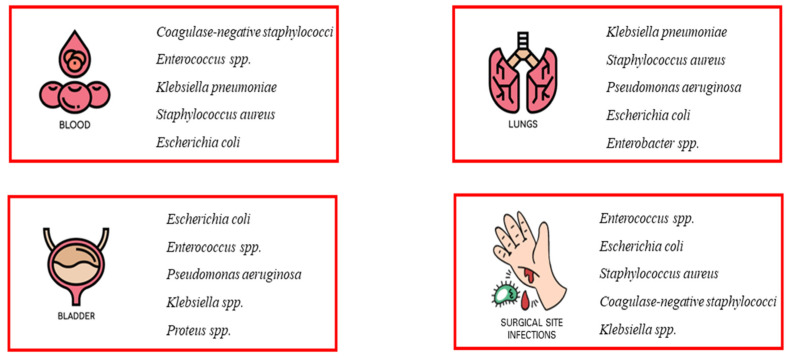
Most common nosocomial infections. The most common infections associated with healthcare facilities include bloodstream infections (BSIs), catheter-associated urinary tract infections (CAUTI), surgical site infections (SSI), and ventilator-associated pneumonia (VAP). The most common pathogens causing nosocomial infections are also listed.

As early as the 20th century, during three influenza pandemics, it was observed that bacterial co-infection was very common in people with an underlying viral infection and exposed the patient to a high risk of deterioration [1,2]. Several studies have shown that most of the deaths that occurred in all three influenza pandemics (Spanish flu of 1918, Asian flu of 1951, and Hong Kong flu of 1968) were not due to the effects of hypervirulent viruses, but rather to secondary bacterial infections responsible for severe and rapidly progressive pneumonia [3,4,5,6]. Nosocomial infections (NI) are a common cause of increased morbidity and mortality in hospitalized patients [7]. In general, infections are classified as community acquired or hospital acquired, and this classification is important in guiding treatment decisions [5,8,9]. Nosocomial infections include all infections that first occur after 48 h of admission or within 3 days of discharge or up to 30 days after surgery [5,9]. Some nosocomial infections, such as catheter-associated urinary tract infections (CAUTI), central line-associated bloodstream infections, and ventilator-associated pneumonia (VAP), compromise patient safety and have been associated with a higher risk of shock and respiratory failure, prolonged hospital length of stay, and increased morbidity and mortality [5]. These infections include occupational infections, which have an equally important impact on healthcare workers [10]. Nosocomial pathogens include bacteria, viruses, and fungi, and the most common infections are bloodstream infections, catheter-associated urinary tract infections (CAUTI), surgical site infections, and ventilator-associated pneumonia [11,12,13] (Figure 1). The most common fungal pathogens leading to hospital-acquired infections are *Candida* spp. and *Aspergillus* spp.; the most common bacterial pathogens are coagulase-negative staphylococci, *Klebsiella pneumoniae*, *Enterococcus* spp., *Staphylococcus aureus*, and *Escherichia coli*; and the most common viral pathogens are influenza and the respiratory syncytial virus [14,15]. These infections occur in both developed and developing countries and account for 7% and 10% of all infections, respectively [16]. In addition, there are an estimated 8.9 and 1.7 million nosocomial infections per year in European and American hospitals, respectively, and the global prevalence is likely to be much higher [17,18].

The emergence of difficult-to-treat multidrug-resistant bacterial infections in healthcare settings is another NI-related adverse event that affects patient safety [19]. The COVID-19 pandemic is an infectious disease caused by a newly discovered coronavirus called SARS-CoV-2 (severe acute respiratory syndrome coronavirus 2) [20]. The emergence and rapid spread of coronavirus disease 2019 (COVID-19 pandemic) placed extraordinary pressure on the healthcare system [21]. The additional burden of COVID-19 care had a negative impact on HAI rates and infection clusters within hospitals. This was despite increased infection prevention and control measures during the pandemic [22]. In this context, it is known that the COVID pandemic had both positive and negative effects on HAIs [5]. Among the positive effects were the adoption of distancing measures, the use of personal protective equipment, and the recommendation to wash hands on a regular basis. In contrast, the negative impact on HAIs has been the overuse of antibiotics, which have been routinely used to treat COVID infections even in the absence of bacterial co-infections [23]. However, the increased focus on infection prevention practices did not offset the additional burden of the pandemic on infection prevention resources [22]. While bacterial infections such as *S. Pneumoniae*, beta-hemolytic streptococci, *H. Influenzae*, and *S. aureus* were significant in almost all influenza deaths, the incidence of bacterial co-infection with coronaviruses is poorly defined. Although previous studies have reported that secondary infections significantly reduce survival in patients with COVID-19, the distinction between secondary infections present on admission and hospital-acquired infections is not always clear in these reports [5,24,25]. Therefore, the purpose of this review is to evaluate the literature on the incidence and outcomes of bacterial co-infections in patients with COVID-19. We also highlight the importance of the rational use of antibiotics in patients with COVID-19 (e.g., the clinical criteria to be used to determine which of these patients should receive empiric antibiotic therapy) and the possibility of using alternative antimicrobial agents to counter the emergence of multidrug-resistant bacteria causing healthcare-associated infections in patients with COVID-19.

### 1.1. The Impact of Coronavirus Disease 2019 (COVID-19)

**Figure 2:**
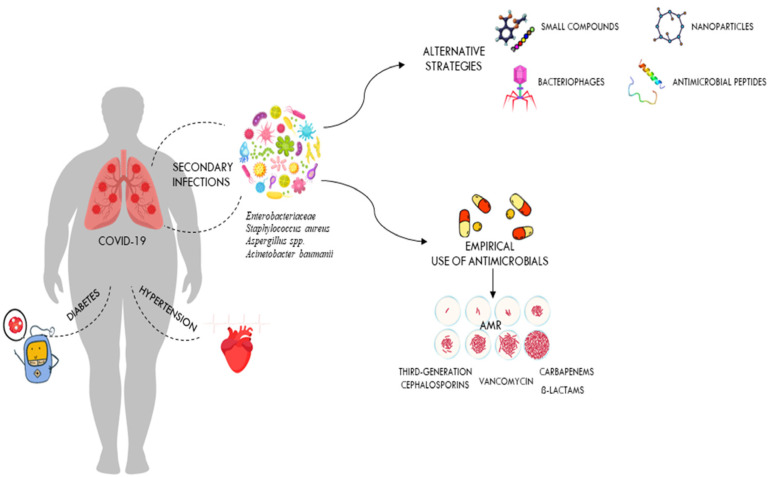
Secondary infections in critically ill patients hospitalised with COVID-19. The figure shows the impact of comorbidities on COVID-19 outcomes and how antibiotic overuse leads to the development of drug-resistant pathogens. It also shows alternative therapies that could be used against these drug-resistant pathogens.

Historically, coronaviruses were known as a family of viruses that usually caused mild colds in people, but outbreaks of severe acute respiratory syndrome (SARS), first identified in China in 2002–2003, followed by the Middle East respiratory syndrome coronavirus (MERS-CoV) in Saudi Arabia in 2012 and most recently coronavirus disease-2019 (COVID-19), show that these viruses can also cause severe illness and death [20,26]. The COVID-19 pandemic is an infectious disease caused by severe acute respiratory syndrome coronavirus 2 (SARS-CoV-2) that was reported in Wuhan, China, in December 2019 and spread very rapidly around the world [20]. This led the WHO to define the epidemic as a pandemic on 11 March 2020. Since the beginning of the COVID-19 pandemic, more than 760 million confirmed cases of COVID-19 and more than 6.8 million deaths have been reported to the WHO worldwide [27]. It should be noted, however, that the spectrum of disease severity in patients infected with SARS-CoV-2 ranges from asymptomatic carrier status to acute respiratory illness to very critical illness requiring admission to intensive care [28]. The severe disease caused by SARS-CoV-2 infection often progresses to multi-organ failure and results in an increased mortality rate among these patients [29]. Apoptotic pathways, increased levels of inflammatory mediators, vascular dysfunction, tissue damage (development of oedema), and infiltration of inflammatory cells into organs contribute to the pathogenesis of COVID-19 [30]. The severity of COVID-19 depends on many factors, including the age and immune status of the infected individuals [30]. Since all humans are susceptible to SARS-CoV-2 infection, but the severity of the disease varies significantly between populations, it is clear that multiple factors may influence the outcome of COVID-19 [31]. The differences in inflammatory and immune responses that influence the clinical outcome of COVID-19 are due to the degree of interaction between different environmental, viral, and host factors [31,32]. They are also related to co-morbidities [33,34]. In particular, respiratory failure associated with COVID-19 is more common in the elderly and in those with pre-existing co-morbidities such as cardiovascular disease, obesity, chronic kidney disease, and diabetes (Figure 2). These patients need immunomodulatory therapy which, together with mechanical ventilation, further increases the risk of HAIs [35]. Patients with severe SARS-CoV-2 infections often require hospitalisation and a prolonged stay in an intensive care unit (ICU), which increases the likelihood of developing a bacterial co-infection [36]. Numerous reports have shown that catheterisation, mechanical ventilation, and tracheostomies are important risk factors for nosocomial infections in intensive care units, which can occur by direct or indirect transmission between health care workers, patients, and medical devices [37,38,39]. It has also been suggested that early antibiotic use in patients with COVID-19, as recommended in the first COVID management guidelines, increases the risk of secondary infections from antibiotic-resistant nosocomial bacteria [5,40]. In addition, as secondary infections are known to have a high mortality rate in patients, it is important to establish the role of these infections in the outcome of COVID-19 patients [41,42].

### 1.2. The Role of Coinfections in Patients with COVID-19

Although there are several studies in the literature reporting secondary infections in patients with COVID-19, the role of these infections in COVID-19 disease requires further investigation [25,43,44]. A literature review was undertaken from September 2020 to March 2023, using the following search terms: “nosocomial infection”, “SARS-CoV-2”, and “COVID-19”, in the PubMed databases. Nine studies were included in this review, including three retrospective studies. Only articles written in English and published in peer-reviewed journals were reviewed. It is not always clear whether these data refer to secondary infections present at the time of admission or those acquired in the hospital. A comprehensive literature review on bacterial co-infections at the time of hospitalisation in patients with COVID-19 was conducted by Westblade et al. [26]. In almost all the studies reviewed by the authors, the rates of co-infection were low. Bacterial infections were present in less than four percent of admissions [26,45]. The majority of these patients were treated with empirical antibacterial therapy, even in the absence of a secondary bacterial infection [26,46]. The significant disparity between the percentage of COVID-19 patients admitted to hospital with antimicrobial treatment and the percentage of patients with an established bacterial infection demonstrates the significant unnecessary use of antimicrobials in COVID-19 patients [45,47]. The inappropriate use of antibiotics at the start of the COVID 19 pandemic was mainly determined by the experience of the previous influenza pandemic, in which bacterial pneumonia resulting from influenza was a major cause of morbidity and mortality [48,49,50]. In order to reduce the risk of ventilator-associated pneumonia in the intensive care unit, most COVID-19 patients were treated with broad-spectrum antibiotics, including third-generation cephalosporins, quinolones, and carbapenems (Figure 2) [51].

However, broad-spectrum empirical therapy may have limited effectiveness, particularly in nosocomial infections, due to the high level of resistance of bacteria that cause life-threatening infections [9]. The lack of effective therapies to treat a new infection is another reason for inappropriate use of antibiotics in patients with COVID-19 (Figure 2) [52]. In a study conducted in the United Kingdom, Adler and colleagues found that bacterial coinfections in COVID-19 patients were rare, in contrast to those in patients with influenza [45]. In contrast, a retrospective study by Bergman et al. showed a similar prevalence of early bacterial co-infections in critically ill patients hospitalised with COVID-19 or influenza [53]. The authors concluded that previous studies reporting a low prevalence of bacterial infections in seriously ill COVID-19 patients should be interpreted cautiously [53]. Another multi-centre retrospective cohort study conducted by Vaughn and colleagues on COVID-19 cases in the USA showed that the majority of patients received empirical antibacterial therapy even though no bacterial infection was present at the time of admission [54]. However, as knowledge of the mode of transmission, incubation period, mechanisms of viral infectivity, and host-associated pathophysiology of COVID-19 has increased, the way clinicians have managed COVID-19 patients has changed [55]. Although previous observational studies have reported that advanced age and other comorbidities, such as chronic kidney disease and diabetes, are risk factors for developing bacterial co-infections in COVID-19 patients, it quickly became clear that these infections can also occur in patients without pre-existing conditions (Figure 2) [56,57]. Although leukocytosis, neutrophilia, and elevated procalcitonin levels are often associated with patients with bacterial co-infections, none of these parameters have sufficient sensitivity and specificity or a positive predictive value to accurately diagnose bacterial co-infections as stand-alone tests [26]. While bacterial co-infections are rare in patients with active COVID-19 on admission, they are common in patients admitted to intensive care units, particularly those requiring prolonged mechanical ventilation (PMV) [58]. In most of these studies, the proportion of secondary infections observed in COVID-19 patients admitted to the ICU ranged from 6% to 28% of patients requiring PMV [26,59]. Most of the COVID-19 patients reported in these studies had been symptomatic for several days before admission and had spent several hours in hospital before intubation [5,60]. In addition, most of these patients, who generally required admission to intensive care and mechanical ventilation, had comorbidities such as hypertension, diabetes, and chronic kidney disease, which increased the risk of severe COVID-19 disease with increased morbidity and mortality (Figure 2) [61]. Bacterial co-infection has been reported to be a major risk factor for mortality in COVID-19 ICU patients and may be due to the high prevalence of invasive procedures and devices (i.e., frequent use of catheters, including endotracheal and arterial catheters) in these patients [62]. In addition, the likelihood of developing bacterial pneumonia and bacteraemia in these patients increases with the number of days spent in intensive care [24,62]. These patients can develop an inflammatory disease that may increase the permeability of the intestinal lining and may allow bacteria to enter the bloodstream. In addition, gut dysbiosis causes inflammatory dysfunction, which can also lead to a severe form of COVID-19 and may be responsible for COVID-19-related death, especially in obese individuals and diabetics [63,64]. Bacteraemia is the second most common infection after bacterial pneumonia in COVID-19 intensive care patients [43]. In COVID-19 ICU patients, bloodstream infections (BSIs) are often caused by bacteria that are resistant to antibiotics and are associated with a high mortality rate [62].

### 1.3. Multidrug-Resistant Bacterial Infections in COVID-19 Patients Admitted to Intensive Care Units

As shown in Figure 2, the presence of comorbidities is often associated with the severity of COVID-19 infection. Overuse of antibiotics in critically ill patients admitted with COVID-19 leads to development of drug-resistant pathogens. The most common bacterial species isolated from patients in a COVID-19 ICU are Gram-negative isolates such as *A. baumannii* and *K. pneumoniae*, while high proportions of MRSA and VRE were observed among the Gram-positive isolates [65,66,67]. Numerous reports indicate that multidrug-resistant *K. pneumoniae* is one of the most important infectious agents isolated from COVID-19 ICU patients [68,69]. In these patients, co-infection with this pathogen causes a wide range of diseases, including pneumonia, urinary tract infections, bloodstream infections, and sepsis [69]. It is also the most common bacterial species isolated from non-COVID-19 ICU patients [7]. Co-infections with carbapenemase-resistant *Klebsiella pneumoniae* are very difficult to treat and have been associated with deterioration in the overall health status of COVID-19 ICU patients [69]. Modification of drug binding sites, efflux pumps, biofilm formation, and transposon acquisition of resistance genes are key components of the major mechanisms of antibiotic resistance in MDR *K. pneumoniae* [70]. Several previous studies in many countries have shown that *A. baumannii* is the most common cause of respiratory infections in COVID-19 patients [71,72]. Co-infection with *A. baumannii* MDR significantly increases morbidity and mortality, especially in COVID-19 patients in intensive care [72]. In addition, this bacterium has been implicated in several outbreaks in healthcare facilities [23,73]. Inappropriate use of personal protective equipment, poor adherence to hand hygiene protocols, and irresponsible use of antibiotics are among the main factors contributing to the outbreak of this nosocomial MDR organism [73]. As with *K. pneumoniae*, several studies worldwide have reported that *A. baumannii* strains with complete resistance to all tested antibiotics except colistin are increasingly being isolated from blood samples collected from ICU patients [8,71]. *E. coli* and *P. aeruginosa* were the other most common bacterial species in ICU patients, although different prevalence and antimicrobial resistance profiles of bacterial co-infections in COVID-19 patients have been reported worldwide [74,75].

Although rare at the time of admission, bacterial infections, particularly those caused by *Pseudomonas aeruginosa*, *Klebsiella pneumoniae*, and *Staphylococcus aureus*, are common during prolonged hospital stays [76]. Previous studies have shown that most patients admitted with COVID-19 do not require initial antibacterial therapy, which is only indicated in critically ill or severely immunosuppressed patients or those with diagnostic tests compatible with bacterial pneumonia [26,48,52]. Blood and respiratory tract cultures may be considered in patients with severe disease who are admitted to intensive care with intubation for respiratory failure. In these patients, empirical antibacterial therapy with beta-lactam agents should be considered [26,52]. A reassessment to discontinue this therapy if microbiological results are negative or to initiate targeted antibacterial therapy according to the identified pathogen should be performed after 48–72 h, according to the American Thoracic Society (ATS) guidelines [77]. *S. aureus*, *S. pneumoniae*, and *H. influenzae* are the most common respiratory and bloodstream bacterial pathogens causing co-infection in COVID-19 patients [26]. Other bacteria involved in this infection, although less common, are MRSA and *P. aeruginosa* [78]. A combination of beta-lactams and macrolides is used to treat these co-infections [78]. Although guidelines recommend the use of combination therapy over monotherapy to avoid the risk of selecting resistant strains, the former gives better results than the latter when given to patients infected with atypical pathogens such as *L. pneumophila* [26]. Anti-MRSA therapy should be discontinued if this microorganism is not isolated from nasal swab cultures of critically ill ICU patients [78]. Pneumonia and bloodstream infections (BSIs) were the most common causes of nosocomial bacterial infections in COVID-19 ICU patients. Major risk factors for nosocomial bacterial infections in COVID-19 patients were hypoxia on admission and the need for mechanical ventilation and ICU admission within 2 days [5]. Coagulase negative staphylococci, *Enterococcus* spp., *Klebsiella pneumoniae*, *P. aeruginosa*, and *S. aureus* were identified as the main pathogens responsible for nosocomial bacterial infections in COVID-19 patients [23]. COVID-19 patients with nosocomial bacterial infections had worse outcomes than those without nosocomial bacterial infections [79]. The former had almost twice the mortality rate of those without nosocomial bacterial infections [80]. Numerous studies worldwide have reported that COVID-19 patients requiring prolonged hospitalisation and mechanical ventilation develop multi-resistant nosocomial infections caused by carbapenemase-resistant Gram-negative bacteria, including *K. pneumoniae*, *A. baumanii*, *Enterobacter cloacae*, and *E. coli* [66,81]. The rapid and widespread increase in carbapenem-resistant Gram-negative infections in COVID-19 patients has been attributed to noncompliance with standard and contact precautions, overworked and inexperienced ICU staff, and a lack of screening for CRE to prevent patient-to-patient transmission [82]. These antimicrobial-resistant infections are difficult to treat and lead to longer hospital stays and higher healthcare costs. The excessive and non-compliant use of antimicrobial agents (e.g., patients taking the wrong or unnecessary antibiotics) causes drug-sensitive bacteria to be killed, while drug-resistant strains persist. These then multiply and accelerate the growth of antimicrobial resistance [83].

### 1.4. The Global Threat of Antibiotic Resistance

The discovery of antibiotics over 90 years ago was a turning point in the history of medicine, saving of millions of lives. However, today, many decades after the introduction of the first antibiotic to treat bacterial infections, we are back to square one: bacterial infections are once again a threat. The overuse and misuse of antibiotics around the world has accelerated the rate at which bacteria develop resistance to the antibiotics designed to kill them, thereby reducing the effectiveness of these drugs. Furthermore, the ever-increasing use of antibiotics in agriculture, often unnecessarily, exacerbates this challenge, leading to increased antimicrobial resistance in humans [57]. The AMR crisis is also attributed to the pharmaceutical industry’s lack of new drug developments due to limited commercial attractiveness and challenging regulatory requirements. According to a report by the UN Ad Hoc Interagency Coordinating Group on Antimicrobial Resistance, if left unchecked, AMR could kill 10 million people a year by 2050 [27].

### 1.5. New Strategies to Tackle AMR

As the search for new classes of antibiotics is bleak and only a few antibiotics may reach the market in a few years, alternative strategies must be developed and diagnostics must be improved to prevent the further spread of drug resistance. Several promising alternatives have been proposed to combat the growing threat of antibiotic-resistant bacteria [84]. These include inorganic nanoparticles, bacteriophages, antimicrobial enzymes, peptides, and small molecules [85]. Inorganic nanoparticles (NPs) act by causing pores in the cell membrane, leakage of the cytoplasm, and disruption of the electron transport chain. Nanoparticles of silver or zinc oxide have been used successfully to combat pathogenic bacteria and fungi [85]. Although inorganic NPs have promising potential for many industrial and commercial applications, including medicine, further studies are needed to determine their effects on ecosystems and human health. Although the use of bacteriophages as antibacterial agents dates back to the early 1900s, phage therapy was eclipsed by the discovery of antibiotics [86]. However, in recent years, there has been a resurgence in interest in bacteriophage therapy as a result of the increase in antibiotic resistance [87]. In this context, the use of bacteriophages as new experimental drugs for the treatment of patients who do not respond to the available antibiotics has been approved by the FDA in a number of cases [88,89]. A phage cocktail, consisting of several phages that infect different types of pathogens, has been successfully used to treat life-threatening infections in humans [90]. It is therefore possible that phage therapy could be of great utility in the fight against secondary infections in critically ill patients with COVID-19. However, to support the routine clinical use of phage therapy, further clinical research studies are needed [91]. Phage lysins, also known as “enzybiotics’, are bacteriophage-encoded lytic enzymes capable of bacterial cell wall degradation [92]. The lytic activity of lysins against MRSA, VRSA, and *S. pneumoniae* infections has been demonstrated in several studies [93]. Of particular interest is the synergy between antibiotics and phage lysins in the treatment of pneumococcal bacteraemia caused by multi-resistant *Streptococcus pneumoniae* [94]. The antimicrobial activity of various small molecules targeting different cellular structures, such as cell wall lipid intermediates, the cytoplasmic membrane, and bacterial RNA polymerase, has been reported in numerous articles [95,96]. The ease with which they can be synthesised and modified makes these small molecules promising candidates for the treatment of antibiotic-resistant infections.

## 2. Discussion

While it is easy to classify an infection as community acquired if it occurs outside a healthcare setting or hospital acquired if it occurs 48 h after a hospital stay, it is much more difficult to distinguish between concomitant and superimposed infections in the reviewed literature [5,26]. The distinction between coinfection and secondary infection/superinfection is also important given that the first guidelines for managing COVID-19 recommend early empiric antibiotic treatment (first hour) in all suspected COVID-19 cases, extrapolating the behaviour of SARS-CoV-2 to that of influenza H1N1 [5]. Given that direct mucosal damage by the virus and dysregulation of the immune response to viral pathogens predisposes patients to bacterial infections and increased mortality, the majority of COVID-19 patients received antibiotics regardless of whether a bacterial infection had been diagnosed [97]. Furthermore, admission to intensive care and long hospital stays are important risk factors for bacterial infection and are associated with poor outcomes. Other risk factors significantly associated with MDR infections in COVID-19 patients include previous colonisation, dialysis, and comorbidities. While the incidence of bacterial co-infections in COVID-19 cases is low, secondary infections are the leading cause of death in immunocompromised COVID-19 patients admitted to intensive care [43,98]. In this setting, *P. aeruginosa*, *Klebsiella pneumoniae*, and *S. aureus* are the most commonly isolated microorganisms causing nosocomial pneumonia [26]. Previous studies have shown a high prevalence of multidrug-resistant bacterial pneumonia and bacteraemia in COVID-19 intensive care patients, particularly during mechanical ventilation [5,26,99]. Improper use of personal protective equipment and poor compliance with hand hygiene protocols are other important factors contributing to the spread of these nosocomial MDR organisms in COVID-19 intensive care patients. It is now clear that the excessive and inappropriate use of antibiotics during the global coronavirus pandemic had a negative impact on the rate of acquisition of bacterial resistance, exacerbating the problem of antimicrobial resistance [100]. Overall, the rate of drug-resistant bacterial infections caused by *Streptococcus pneumoniae*, *Klebsiella pneumoniae*, *Haemophilus influenzae*, *Escherichia coli*, *Staphylococcus aureus*, *Pseudomonas aeruginosa*, and *Acinetobacter baumannii* increased with the increased use of antibiotics in COVID-19 admissions [57,101]. AMR infection results in longer hospital stays and consequently higher healthcare and second-line drug costs. In order to preserve the effectiveness of the most important antibiotics, it is essential to follow specific guidelines when prescribing antibiotics. These guidelines include appropriate diagnosis of the microbial infection, assessment of the severity of the infection, and prescription of appropriate antibiotics. The best way to meet these guidelines is to develop rapid diagnostic technologies that can differentiate viral from bacterial infections, thereby avoiding unnecessary antimicrobial treatment. These diagnostic technologies should also be able to determine antimicrobial susceptibility and help prescribe appropriate narrow-spectrum drugs. Despite ongoing efforts to reduce antibiotic overuse, prescribing antibiotics remains unavoidable, especially for secondary bacterial infections that can lead to invasive infections if left untreated [57]. Therefore, the implementation of two strategies appears crucial to effectively treat bacterial infections and prevent the further spread of drug resistance, these are (1) rethinking the principles of antimicrobial stewardship to prevent the emergence and transmission of drug-resistant organisms in healthcare facilities and (2) establishing guidelines for the safe and effective use of antimicrobials, such as bacteriophages, antimicrobial peptides, and nanoparticles, and phage lysins for the treatment of critically ill COVID-19 patients who do not respond to available antibiotics.

## Data Availability

Not applicable.
